# A Cross-sectional Study for Assessment of Untreated Dental Caries and Its Consequences among Slum-dwelling Children

**DOI:** 10.5005/jp-journals-10005-1402

**Published:** 2017-02-27

**Authors:** Charu Marya, Sakshi Kataria, Ruchi Nagpal, Sukhvinder S Oberoi, Chandan Dhingra, Dimple Arora

**Affiliations:** 1 Head, Department of Public Health Dentistry, Sudha Rustagi College of Dental Sciences & Research, Faridabad, Haryana, India; 2 Postgraduate, Department of Public Health Dentistry, Sudha Rustagi College of Dental Sciences & Research, Faridabad, Haryana, India; 3 Reader, Department of Public Health Dentistry, Sudha Rustagi College of Dental Sciences & Research, Faridabad, Haryana, India; 4 Reader, Department of Public Health Dentistry, Sudha Rustagi College of Dental Sciences & Research, Faridabad, Haryana, India; 5 Senior Lecturer, Department of Public Health Dentistry, Sudha Rustagi College of Dental Sciences & Research, Faridabad, Haryana, India; 6 Senior Lecturer, Department of Public Health Dentistry, Sudha Rustagi College of Dental Sciences & Research, Faridabad, Haryana, India

**Keywords:** Dental caries, Dental public health problem, Primary oral health care.

## Abstract

**Introduction:**

Dental caries affects humans of all ages throughout the world and remains the major dental public health problem among children globally.

**Materials and methods:**

A cross-sectional study was conducted using the cluster sampling technique to obtain the required sample size of 400 children from urban slum population of Faridabad, Haryana, India. Data were collected using World Health Organization dentition status 1997 criteria and PUFA/pufa index.

**Type of study:**

Cross-sectional study.

**Results:**

Among 6- to 12-year-age group, 68.5% subjects had one or more decayed deciduous teeth, of which 65.3% subjects had one or more pufa score, and 26.4% subjects were having one or more decayed permanent teeth, of which 16.7% subjects were having one or more PUFA score. The overall caries prevalence was 79.64% and overall prevalence of one or more than one PUFA+pufa was reported in 69.2% subjects.

**Conclusion:**

The findings of the study showed the relevance of PUFA/pufa index to address the neglected problem of untreated caries and its consequences. The study also suggests the importance for implementation of the primary oral care programs for this deprived population.

**How to cite this article:**

Marya C, Kataria S, Nagpal R, Oberoi SS, Dhingra C, Arora D. A Cross-sectional Study for Assessment of Untreated Dental Caries and Its Consequences among Slum-dwelling Children. Int J Clin Pediatr Dent 2017;10(1):29-33.

## INTRODUCTION

Oral diseases represent a global public health problem, yet are marked by social inequality; the burden is substantially higher among poorer and disadvantaged populations in both developed and developing countries. Thus, socioeconomic status is an important determinant of oral health.^1^

Dental caries is a worldwide phenomenon. It affects humans of all ages throughout the world and remains the major dental health problem among children globally.^2^

Rapid urbanization is a global phenomenon. Increasing urbanization is emerging as the most pervasive and dominant challenge. This, accompanied by sustained population growth due to large-scale migration from rural areas to urban centers, leads to mushrooming of slum settlements in all cities and towns in India. The State of the World’s Children 2012: Children in an Urban World by UNICEF (2012)^3^ reported that slum children in urban areas across the world, including India, happen to be the most deprived and their sufferings are often obscured by broad statistical averages. They are increasingly facing the negative consequences of rapid urbanization, high density, acute shortage of housing and basic civic amenities, degradation of environment, traffic congestion, pollution, poverty, unemployment, crime, and social unrest.^4^

This group of children and adolescents in urban areas are likely to have greater access to alcohol, illegal drugs, higher number of cariogenic intakes per day, high frequency of sweet food and candies, eating foods before sleeping than their counterparts in urban areas. There is also lack of mother’s care for a long period.^3^

The booming industries in the Faridabad during the 1970s and 1980s and the growth of its economy have contributed to the growth of slums. Large numbers of laborers from different parts of the country have migrated in groups to the city in search of opportunities and settled on available vacant lands.^5,6^

Till date, there is no baseline data available on the prevalence of dental caries of these underprivileged children residing in urban slum areas in different parts of India. However, both in developing and economically developed countries, there are groups from a lower socioeconomic background with a higher risk of severe caries. The differences in the epidemiological situation within countries and the failure of decayed-missing-filled (DMF) index to provide information on the clinical consequences of untreated dental caries, such as pulpal abscess, which may be more serious than the carious lesions themselves, resulted in the development of new tools for the assessment of caries severity.

Hence, an attempt was made in this present cross-sectional study to assess untreated dental caries among Indian slum children using pufa index.

## MATERIALS AND METHODS

### Ethical Clearance

The study protocol was approved by Institutional Ethical Review Committee of Sudha Rustagi College of Dental Sciences and Research, Faridabad, Haryana, India. A written informed consent to carry out the study was obtained from the parents/legal guardian of each participant before carrying out the examination which was in accordance with the World Medical Association Declaration of Helsinki. Verbal consent was obtained from the participants.

### Study Population

A cross-sectional study was carried out in the months of October and November in 2014, in the urban slum areas of Faridabad, Haryana. Based on the inclusion criteria, 400 children, aged 5 to 15 years, were selected.

Inclusion Criteria

- People residing in the urban slum areas of Faridabad district
- Children in the age group of 5 to 15 years

The referral services were also provided for meeting the dental needs of urban slum children. There was no refusal rate as they were provided with toothpaste and toothbrush as incentives. Dental health education was also provided at the end of the examination.

### Sampling Methodology

The cluster sampling methodology was used for the selection of the study population. A pilot study was done on 60 subjects, where the prevalence of dental caries was found to be 85%.

N = Zα^2^ {p(1 - p)}/L^2^

The estimated sample size was 196, which was further multiplied by a correction factor of 2 (by default, the intraclass correlation coefficient could not be calculated as there are limited studies evaluating PUFA), to compensate for the design effect of cluster sampling; the final sample size was determined as 392 which was rounded off to 400 children.

The Faridabad district is divided into three blocks: Old Faridabad, New Industrial Township (NIT), and Ballabhgarh. In all, there are 67 identified slum clusters in the city. But the data regarding the distribution of clusters in these individual blocks were not available. Due to high migration rate, this urban slum population was also ill-defined. Hence, to overcome both of these problems, quota sampling was adopted. In this study, the quota was decided on the basis of overall population density of individual blocks. Out of total 400 subjects, 50% of the subjects, i.e., 200 were selected from NIT block as it had maximum population density; 30% of the subjects, i.e., 120 were selected from Ballabhgarh; and 20% of the subjects, i.e., 80 were selected from Old Faridabad block as it had the minimum population density.

The corresponding map of the individual blocks was obtained. The head of the slum area was approached a day prior to the examination, for general guidance. The starting point of the cluster, the first home to be visited by an examiner, was selected randomly from central part of slum. All the children residing in the house were identified and screened for eligibility. Households were visited in a row following specific rules; i.e., the direction of movement/travel was counterclockwise till the desired sample size for the concerned individual block was achieved. The same methodology was followed in the other two blocks also.

This quota sampling was less costly, less time-consuming, and the nonresponse was also avoided as the examiner continued beyond nonresponding households until he/she obtained enough responding ones to fulfill the quota. Furthermore, the technique was unbiased as the starting point along the path of travel was determined randomly.

## CLINICAL EXAMINATION

### Calibration of Examiner

All measurements were performed by a single calibrated dentist. Internee from the same institute served the purpose of a recording clerk.

The examiner after selecting the subjects brought them to the sheltered place. The oral examination was performed by the examiner under open natural daylight, with children sitting on chair with back rest. The instruments used were plane mouth mirrors and community periodontal index probe.

The dentition status was assessed using World Health Organization (1997) criteria along with the PUFA/pufa index for severity of dental caries. Reliable results were seen with kappa values of 0.82 and 0.80 respectively. The PUFA is an index used to assess the presence of oral conditions resulting from untreated caries.^9^ It scores the presence of either a visible pulp, ulceration of the oral mucosa due to root fragments, a fistula, or an abscess. Lesions in the surrounding tissues that are not related to a tooth with visible pulpal involvement as a result of caries are not recorded. The assessment is made visually without the use of an instrument. Only one score was assigned per tooth. In case of doubt concerning the extent of odontogenic infection, the basic score (P/p for pulp involvement) is given. If the primary tooth and its permanent successor tooth are present, and both present stages of odontogenic infection, both teeth will be scored. Uppercase letters are used for the permanent dentition and lowercase letters used for the primary dentition.

The PUFA/pufa score per person is calculated in the same cumulative way as for the DMFT/dmft and represents the number of teeth that meet the PUFA/pufa diagnostic criteria. The PUFA for permanent teeth and pufa for primary teeth are reported separately. Thus, for an individual person the score can range from 0 to 20 pufa for the primary dentition and from 0 to 32 PUFA for the permanent dentition. The prevalence of PUFA/pufa is calculated as percentage of the population with a PUFA/ pufa score of one or more. Laminated pictures were used as a reference for PUFA/pufa scoring.

### Duplicate Examination

A subsample of 10% was clinically examined by another examiner for dentition status and PUFA/pufa, who was blinded to the earlier recordings.

### Statistical Analysis

The data were analyzed using the Statistical Package for the Social Science software (version 20). Mean, standard deviation, frequencies, and percentages were calculated. The data were presented in the form of tables.

## RESULTS

Out of 400 subjects, 199 (49.8%) were males and 201 (50.2%) were females. Tables 1 and 2 show prevalence of caries and prevalence of PUFA/pufa based on age groups respectively. The results of the present study show that among the study population, i.e., 5- to 15-year age group, 55.5% subjects were having one or more decayed deciduous teeth, whereas 31.75% subjects were having one or more decayed permanent teeth. Among the study population, 52.7% subjects were having one or more pufa score, and 22.7% subjects were having one or more PUFA score. The overall caries prevalence was reported by 76% subjects (number of subjects having either decayed permanent or deciduous teeth) and the overall prevalence of one or more than one PUFA+pufa (number of subjects having either pufa or PUFA) was reported among 66.5% subjects.

Tables 3 and 4 show mean caries experience [standard deviation (SD)] and mean PUFA/pufa (SD) respectively. The mean number of decayed teeth among the study population was 2.22 (2.42) while mean number of decayed deciduous teeth was 1.63 (2.42) and mean number of decayed permanent teeth was 0.59 (1.06). The mean pufa/ PUFA among the study population was 1.63 (1.85) while mean pufa was found to be 1.31 (1.86) and the mean PUFA was 0.32 (0.68). The mean “p” among the study population was found to be 0.94 (1.29), mean “u” was found to be 0.20 (0.54), mean caries “f” was found to be 0.02 (0.18) and mean “a” was found to be 0.15 (0.63). The mean “P” among the study population was found to be 0.28 (0.60), mean “U” was found to be 0.02 (0.22), mean caries “F” was found to be 0.00(0.05) and mean “A” was found to be 0.01 (0.11). The overall “untreated caries, pufa ratio” indicates the percentage of decayed component from the study population that has progressed to pulpal involvement. It is calculated using the formula:

(PUFA + pufa) / (D + d) × 100

where D = total number of decayed permanent teeth, d = total number of decayed deciduous teeth. It was found to be 59.14% among the study population.

**Table 1:** **Table 1:** Prevalence of caries (%) based on age groups

		*5 years*				*6-12 years*				*13-15 years*				*5-15 years*			
		*n*		*%*		*n*		*%*		*n*		*%*		*n*		*%*	
Prevalence of decayed deciduous teeth > 0		27		62.7		192		68.5		3		3.89		222		55.5	
Prevalence of decayed permanent teeth > 0		0		0.0		74		26.4		53		68.8		127		31.7	
Overall caries prevalence		27		62.7		223		79.6		54		70.1		304		76	

**Table 2:** **Table 2:** Prevalence of PUFA/pufa (n%) based on age groups

		*5 years*				*6-12 years*				*13-15 years*				*5-15 years*			
		*n*		*%*		*n*		*%*		*n*		*%*		*n*		*%*	
Prevalence of pufa > 0		25		58.1		183		65.3		3		3.89		211		52.7	
Prevalence of PUFA > 0		0		0		47		16.7		44		57.1		91		22.7	
Overall prevalence of PUFA/pufa		25		58.1		194		69.2		47		61.0		266		66.5	

**Table 3:** **Table 3:** Mean caries experience (SD) based on age groups

		*5 years*		*6-12 years*		*13-15 years*		*5-15 years*	
Mean (SD) decayed deciduous teeth		2.09 ± 3.33		1.97 ± 2.38		0.13 ± 0.92		1.63 ± 2.42	
Mean (SD) decayed permanent teeth		0.00 ± 0.00		0.39 ± 0.76		1.64 ± 1.51		0.59 ± 1.06	

**Table 4:** **Table 4:** Mean PUFA/pufa (SD) based on age groups

		*5 years*		*6-12 years*		*13-15 years*		*5-15 years*	
Mean (SD) pufa		1.51 ± 2.00		1.60 ± 1.90		0.13 ± 0.92		1.31 ± 1.86	
Mean (SD) PUFA		0.00 ± 0.00		0.22 ± 0.56		0.84 ± 0.93		0.32 ± 0.68	
Mean (SD) P		0.00 ± 0.00		0.19 ± 0.47		0.77 ± 0.87		0.28 ± 0.60	
Mean (SD) U		0.00 ± 0.00		0.02 ± 0.25		0.03 ± 0.16		0.02 ± 0.22	
Mean (SD) F		0.00 ± 0.00		0.00 ± 0.00		0.01 ± 0.11		0.00 ± 0.05	
Mean (SD) A		0.00 ± 0.00		0.01 ± 0.08		0.04 ± 0.19		0.01 ± 0.11	
Mean (SD) p		1.09 ± 1.43		1.16 ± 1.33		0.05 ± 0.28		0.94 ± 1.29	
Mean (SD) u		0.23 ± 0.57		0.24 ± 0.55		0.05 ± 0.45		0.20 ± 0.54	
Mean (SD) f		0.02 ± 0.15		0.03 ± 0.20		0.00 ± 0.00		0.02 ± 0.18	
Mean (SD) a		0.16 ± 0.57		0.18 ± 0.70		0.03 ± 0.23		0.15 ± 0.63	

## DISCUSSION

Over the last century, a meaningful increase in general health standards has been achieved through better living and working conditions, as well as improved nutrition and environmental aspects like sanitation. However, these improvements have not been extended in a homogeneous way.^1^

The 58th World Health Assembly, urges to ensure adequate and equitable distribution of good-quality health-care infrastructure and human resources.^7^ For the past few years, international caries epidemiology has focused on the development of more sensitive diagnostic criteria for assessment of the initial stages of caries. This is an asset to high-income countries where nonopera-tive and preventive interventions require an index that distinguishes between the different stages of initial caries lesions. However, in deprived communities, where people have little access even to the most basic forms of care, there is a need for a diagnostic index that addresses the advanced stages of untreated caries lesions.

A closer review of the literature shows that by exposing decision makers only to DMFT data leaves them unaware of the high levels of untreated caries lesions, their severity and associated health and quality of life consequences.^8^

The PUFA index was developed in response to that need. The various clinical stages defined by PUFA have different associations with health conditions, providing “a face of the reality” to the prevailing and often ignored oral conditions.^9^ Measurement of change in disease levels is an important component of epidemiology.^10^ Presenting data based on the PUFA index will provide with relevant information, which is complementary to DMFT. Many researchers like Gradella et al,^11^ Figueiredo et al,^12^ and Leal et al,^13^ in their respective studies, have found PUFA/ pufa as universally appropriate in quantifying the consequences of severe tooth decay in all settings. The strict comparisons with other studies are made with caution due to a paucity of similar studies. Due to different age profile of all the studies, the findings cannot be directly compared to that of the present study. Yet some facts can be highlighted about the current scenario of these underprivileged populations.

A study conducted by Gradella et al^11^ showed a high prevalence (62%) of dental caries in the primary dentition among 2- to 4-year-old children. The similar type of results was also seen in the present study with a high prevalence (62.7%) of dental caries among the 5-year age group.

In the same study by Gradella et al,^11^ overall prevalence of pufa was found to be 5%, which is very less as compared to 58.1%, found in the 5-year age group of the present study. This reflects the scenario of health care needs of the population living in the “dark underbelly” of developing country India. These findings can also be supported by the study conducted by Bonanto et al^14^ where they determined the association between oral disease, access to dental care, and social class among 5-year-old preschool children in Brazil. They concluded that oral diseases in the primary dentition and access to dental treatment are affected by social and cultural factors.

The prevalence of pufa score (65.3%) among 6- to 12-year-old age group in the present study was found to be higher in comparison to the studies by Figueiredo et al^12^ (23.7%) and Leal et al^13^ (26.2%) on 6- to 7-year-old age group. This could be justified on the grounds that the permanent molars have been exposed to a longer duration since the time they erupted in the oral cavity at 12-year age as compared to the 7-year age. Hence, they are more prone to carious attack.

Table 4 shows mean PUFA/pufa of the present study. These findings found among 6- to 12-year age group of the present study were nearly similar in comparison to a study conducted by Monse et al^8^ where the mean pufa was reported as 0.1 and 1.0, mean “p” as 2.9 and 0.2, mean “u” as 0.3 and 0.0, mean “f” as 0.1 and 0.0, and mean “a” as 0.1 and 0.0 among 6- to 12-year age group respectively, and also with the study of Figueiredo et al^12^ where mean pufa was found to be 0.4, mean “p” as found to be 0.3, mean “u” as found to be 0.001, mean “f” as 0.08, and mean “a” was found to be 0.01. These were nearly similar to the findings. The same study by Monse et al^8^ reported mean “P” to be 0.1 and 0.8, mean “U” to be 0.0 and 0.1, mean “F” to be 0.0 and 0.1, and mean “A” to be 0.0 and 0.1.

The overall “untreated caries, PUFA ratio” among 6- to 12-year age group in our study was found to be 77.2% which is very high in comparison to the study done by Monse et al^8^ (40 and 41% among 6- and 12-year-olds respectively) and Shanbhog et al^15^ among orphanage children aged 12 to 14 years (21%).

Thus, PUFA when taken along with the DMFT index can serve as an asset describing the burden of untreated cavitated caries lesions at the tooth and surrounding tissue stages, especially in developing countries like India where suppression of deprivation and social inequalities cannot be easily achieved. The access to oral health services is also limited and teeth are often left untreated or are extracted because of pain or discomfort.
